# Exploring orthorexia nervosa risk in high school and university students: associations with perceived social support, body esteem, and diet quality

**DOI:** 10.1007/s40519-026-01884-y

**Published:** 2026-06-20

**Authors:** Marta Plichta, Nanette Stroebele‐Benschop

**Affiliations:** 1 Department of Food Market and Consumer Research, Institute of Human Nutrition Sciences, Warsaw University of Life Sciences (SGGW-WULS), Nowoursynowska St. 159c, 02-776 Warsaw, Poland; 2 Department of Applied Nutritional Psychology, Institute of Nutritional Medicine, University of Hohenheim, Fruwirthstr. 12, 70593 Stuttgart, Baden-Württemberg Germany

**Keywords:** Orthorexia nervosa, Perceived social support, Body esteem, Diet quality, High school students, University students

## Abstract

**Purpose:**

The lives of people with eating disorders (EDs) habitually revolve around food and diet, and they exhibit disturbances in attitudes and feelings about their bodies and appearance. Both aspects are also described in people at risk of orthorexia nervosa (ON). Social support plays an important role in the treatment of EDs, but less is known about the role of social support in ON. Therefore, the study aimed to estimate the levels of ON risk, perceived social support, body esteem (BE), and diet quality among students by biological sex, body mass index (BMI) group, and current education stage and program; and evaluate the associations between the levels of ON risk, perceived social support, BE, and diet quality.

**Methods:**

Data were collected from 2022 to 2025 through a cross-sectional quantitative survey among 261 Polish high school students (aged 16–19 years) and university students (aged 18–23 years). Students completed the Polish version of the Düsseldorf Orthorexia Scale (PL-DOS), the Multidimensional Scale of Perceived Social Support (MSPSS), the Body Esteem Scale for Adolescents and Adults (BESAA), and the Dietary Habits and Nutrition Beliefs Questionnaire (KomPAN). Pearson Chi-squared test, the Mann–Whitney U test, the Kruskal–Wallis rank ANOVA, and quantile regression analysis were used for further analyses.

**Results:**

Females exhibited a significantly higher ON risk than males. University students from nutrition-related majors demonstrated a significantly higher ON risk than those from non-nutrition-related majors. No significant association was found between ON risk and BMI. An increased risk of ON was significantly associated with lower social support from friends, lower BE weight, and a lower intensity of harmful dietary characteristics for health. Meanwhile, higher BE attribution and a higher intensity of beneficial dietary characteristics for health were significantly associated with increased ON risk.

**Conclusions:**

Overall, the findings suggest that ON risk is associated with selected psychosocial characteristics, such as perceived social support and body weight-related self-esteem. These findings highlight the need for greater integration of psychological aspects of eating and body image into school and university education programs, as well as interventions aimed at enhancing support from friends to reduce the risk of ON and EDs.

*Level of evidence*: Level III. Evidence obtained from a cross-sectional quantitative study.

## Introduction

Orthorexia nervosa (ON) is understood as an overvaluation of and excessive preoccupation with one’s eating behaviors and their impact on health [1]. Individuals with ON experience emotional distress, anxiety, and guilt resulting from failure to adhere to self-imposed, rigid dietary rules [2]. They may exclude high-calorie, high-fat, and high-sugar foods from their diet; therefore, dietary restrictions may resemble those of weight-loss dieters or individuals with weight/shape concerns and/or eating disorders (EDs) [1]. There are still considerable controversies regarding the classification of ON because of its similarity to currently recognized EDs, particularly anorexia nervosa (AN) [3]. Consequently, ON has not been formally classified as a distinct mental disorder, neither in the International Classification of Diseases and Related Health Problems, 11th Revision (ICD-11) [4] nor in the Diagnostic and Statistical Manual of Mental Disorders, Fifth Edition (DSM-5) [5]. Researchers assume that ON may occur on the same spectrum as AN, with individuals with ON shifting from an obsession with the quantity of food to an obsession with its quality [6]. Furthermore, an obsession with healthy eating may be a socially acceptable method of weight control for individuals with AN [3]. Originally, the prevailing view in the literature was that people with ON were not preoccupied with their weight and shape, dissatisfied with them, or concerned about gaining weight [1]. A recently published consensus about ON emphasized that the self-esteem of individuals with ON focuses on their ability to follow self-imposed dietary rules to improve their health and/or avoid negative health consequences, unlike individuals with AN, whose self-esteem focuses on weight and shape [2]. However, recent reports involving i.a., university students have shown associations between ON risk and lower self-esteem [7], drive for thinness [8], and body dissatisfaction [8]. This suggests that a preoccupation with body weight and/or shape may also be present among individuals with ON [8]. Researchers further suggest that body dissatisfaction may be a contributing factor to low self-esteem among middle-aged females [9]. Hence, it is worth investigating the association between ON risk and self-esteem related to physical appearance in greater depth.

EDs also appear to be related to social and environmental factors [10]. Adults suffering from EDs experience social anxiety, which may lead to social isolation and loneliness [10]. They may also experience fear of social or performance situations in which they are exposed to possible judgment by others [11]. It has been shown that social support influences the symptoms of EDs [11]. Young females with ED symptoms report lower levels of perceived social support than those without EDs [11]. Similarly, recent studies among university students have shown that ON is also associated with lower perceived social support [12, 13]. Social support, whether actual or perceived, may serve as a buffer that mitigates ED symptoms; therefore, it seems reasonable to examine these aspects in the context of ON [11].

Moreover, individuals with ON tend to exaggerate ideas about foods and their consequences, which can excessively affect attitudes and behaviors related to eating [2]. The diet followed by people with ON is characterized by the strict avoidance or complete elimination of certain foods, or entire food groups perceived as unhealthy or impure [1]. Although the list of affected foods may vary, the gradual nature of dietary restriction is typical [14]. In addition, any violation of self-imposed dietary rules may result in a desire to discipline oneself by intensifying restrictions or fasting [14]. Although dietary restrictions associated with ON are intended to improve health and/or avoid negative health consequences, suggesting that the diet of individuals with ON may be of good quality, it remains unclear whether the successive elimination of foods is associated with a deterioration in diet quality over time [15]. To date, only a few studies have assessed diet quality among individuals at risk of ON, with mixed results [14, 16–19]. However, three of these studies used shortened versions of the ORTO-15 questionnaire to assess ON risk, namely ORTO-11 [18] and ORTO-6 [14, 16]. Studies using ORTO-6 among high school students, university students, and young university staff confirmed a positive association between ON risk and diet quality [14, 16], whereas among adults, no such association was found with ORTO-11 [18]. The remaining studies among adults used the Eating Habits Questionnaire (EHQ) [19] and the Teruel Orthorexia Scale (TOS) [17, 19], with the latter dividing participants into two groups: “Healthy orthorexia—HeOr” identifying individuals interested in healthy eating, and “Orthorexia nervosa—OrNe” referring to individuals with a pathological obsession with healthy eating [17]. One study confirmed a positive association between ON risk and diet quality using both the EHQ and TOS for HeOr and OrNe [19]. A subsequent study showed a positive association only between HeOr and diet quality, whereas for OrNe, this association was negative [17]. In one of the most recent studies among university students comparing ORTO-15 and its shortened versions with ORTO-R and the Polish version of the Düsseldorf Orthorexia Scale (PL-DOS), a higher ON risk, as measured by almost all questionnaires, was associated with better diet quality [20]. However, high school students have not yet been studied using PL-DOS in this context. The preoccupation with one’s diet, health, and body image often starts in adolescence and young adulthood, making the investigation of this age group particularly important. Therefore, this study aimed to: (a) estimate the levels of ON risk, perceived social support, body esteem, and diet quality among high school and university students according to biological sex, BMI groups, and current education stage and program; and (b) evaluate the associations between the levels of ON risk, perceived social support, body esteem, and diet quality. To the best of the authors’ knowledge, no study has yet assessed all of these aspects simultaneously. Conducting research on the risk of ON among high school students constitutes a crucial component of preventive mental health activities for young people. School settings are one of the key environments in which it is possible to systematically monitor young people’s mental health and implement early educational interventions. The following hypotheses were proposed:

### H1

The current education stage and program are related to the level of ON risk.

### H2

A decrease in perceived social support is associated with an increase in the level of ON risk.

### H3

Individuals with a higher level of ON risk exhibit lower levels of self-esteem, especially related to body weight.

### H4

The level of ON risk is associated with higher diet quality.

## Methods

### Study design and participant recruitment

A cross-sectional quantitative survey was conducted from April 2022 to May 2025. The study protocol was approved by the Ethics Committee of the Faculty of Human Nutrition and Consumer Science, Warsaw University of Life Sciences (Resolution No. 46p/2019). Data were collected intermittently at several recruitment points during the study period, depending on institutional accessibility. The invitation to study was addressed to several school heads and faculty deans at the university via e-mail. After institutional approval was obtained, recruitment was coordinated with designated staff members (school teachers, academic teachers, school pedagogues, or school psychologists), who assisted in identifying eligible students within the school grade or year of study and facilitating communication with potential participants. A total of 14 schools were invited to participate, including 2 high schools with a gastronomic education program, 12 with a comprehensive education program, and one university with nutrition-related and non-nutrition-related majors. Both gastronomic high schools and the university agreed to participate, whereas only one comprehensive high school responded positively. After achieving the recruitment aims, no further invitations were distributed. Three high schools and one university located in the Masovian Voivodeship in Poland participated in the end. Participants were recruited during lessons and lectures at the high schools and the university. As a result of this procedure, students who participated had already been pre-screened for willingness to take part through school- or university-mediated selection; therefore, no refusals were observed at the time of data collection. The selection of high schools and the university was nonrandom. The same study protocol, inclusion and exclusion criteria, questionnaire, and procedures were applied across all recruitment points. Exclusion criteria included: (1) no high school or university student status; (2) high schools/university located outside the Masovian Voivodeship; (3) lack of consent to participate in the study; and (4) lack of consent from the parent/legal guardian for the minor to participate in the study. No incentives (financial or school/university credit) were offered for participation. Participation was voluntary. Third- and fourth-grade high school students and first-year university students participated in the study. The age of high school students ranged from 16 to 19 years, while university students were aged 18–23 years. For participants under 18 years of age, parental or legal guardian consent forms were distributed through school staff to students who expressed initial interest in participation. Completed consent forms were collected prior to data collection by school staff. Written assent was obtained from all students before participation. All procedures were conducted in cooperation with school and university staff.

**Fig. 1:**
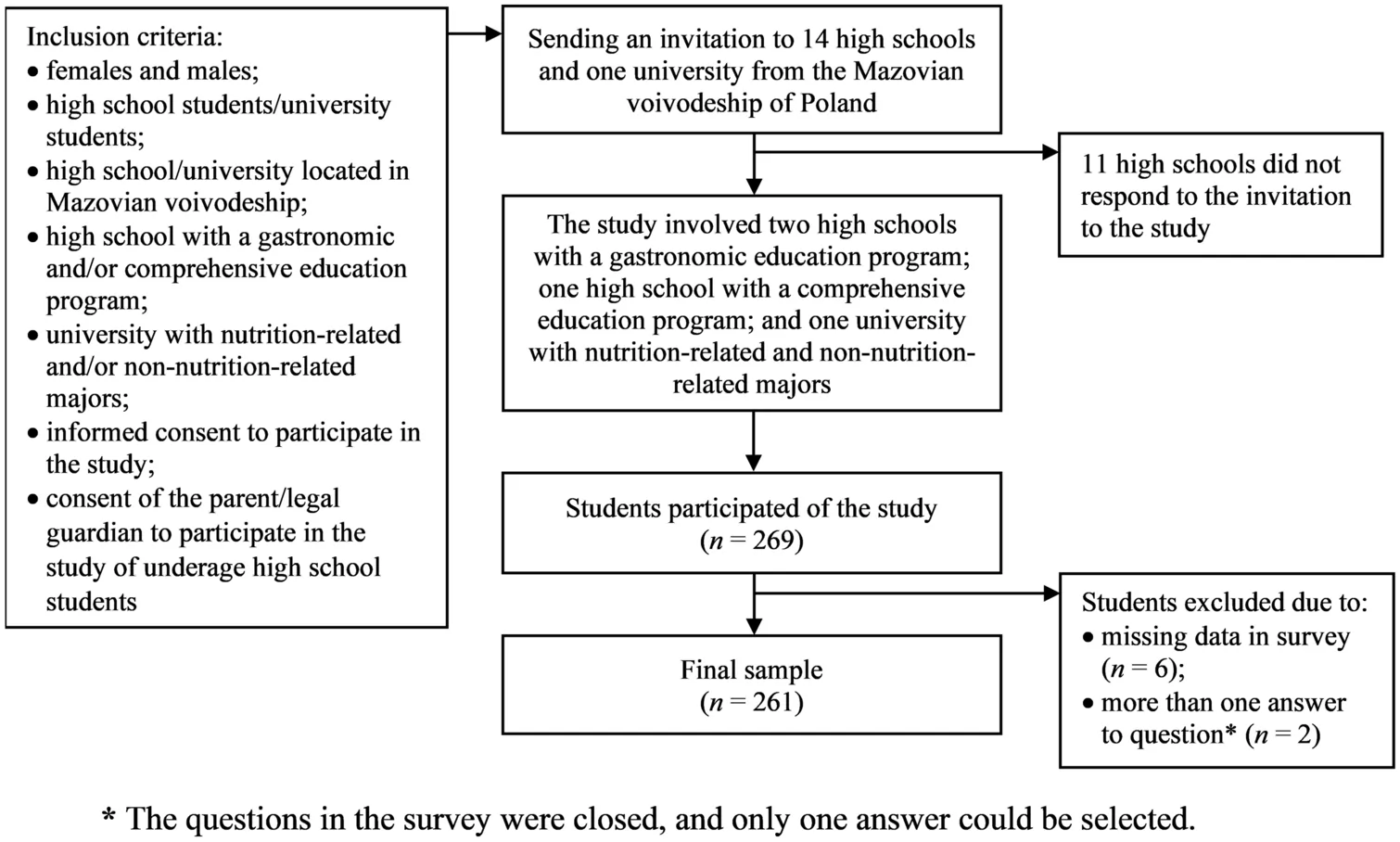
Study design and participant recruitment

The study was conducted using an auditorium survey with a paper-and-pencil personal interview (PAPI) technique. The choice of this research method was based on the possibility of gathering participants in one place at the same time. The selection of the high school and university students was guided by literature reports indicating a higher prevalence risk of ON among individuals learning about nutrition-related issues [21, 22]. Participants were informed about the purpose, procedure, and duration of the survey, as well as the possibility of withdrawing from the study at any stage without any legal consequences. Selected data were pseudonymized to increase the level of personal data security. Participants completed the survey in approximately 25 min. Due to missing data in the questionnaires, six participants were excluded from the database. In addition, two participants were excluded from further analysis because they provided more than one answer to certain questions. The final sample consisted of 261 high school and university students. Figure 1 presents details of participant recruitment.

### Risk of orthorexia nervosa

Orthorexia nervosa was assessed using the validated Polish version of the Düsseldorf Orthorexia Scale (PL-DOS) [23], which was culturally adapted based on the English version of the Düsseldorf Orthorexia Scale (E-DOS) [24]. The PL-DOS consists of 10 items to which respondents answered on a four-point Likert scale ranging from “this applies to me” (4 scores) to “this does not apply to me” (1 score). The PL-DOS includes three subscales: negative feelings (3 items, e.g., “I feel upset after eating unhealthy foods”); social exclusion (3 items, e.g., “I try to avoid getting invited over to friends for dinner if I know that they do not pay attention to healthy nutrition”); and importance of healthy nutrition (4 items, e.g., “I have certain nutrition rules that I adhere to”). The total PL-DOS score ranges from 10 to 40. Scores for the negative feelings and social exclusion subscales range from 3 to 12, whereas scores for the importance of healthy nutrition subscale range from 4 to 16. Higher scores indicate a more pronounced ON. In addition, the questionnaire allows respondents to be categorized into three groups: absence of ON (score < 25), risk of developing ON (score 25–29), and presence of ON (score ≥ 30). Cronbach’s alpha coefficient for the PL-DOS in this study was 0.82.

### Perceived social support

The validated Polish version of the Multidimensional Scale of Perceived Social Support (MSPSS) was used to assess perceived social support [25]. The Polish version of the MSPSS consists of 12 items answered on a seven-point Likert scale: 1—“I strongly disagree”; 2—“I disagree”; 3—“I disagree somewhat”; 4—“I do not have an opinion”; 5—“I somewhat agree”; 6—“I agree”; and 7—“I strongly agree”. The MSPSS includes three subscales reflecting basic sources of support: significant others (4 items, e.g., “There is a special person in my life who cares about my feelings”); family (4 items, e.g., “My family really tries to help me”); and friends (4 items, e.g., “I can talk to my friends about my problems”). Scores were calculated for individual subscales as well as for the entire questionnaire, yielding an overall perceived social support score. The possible score range for the Polish version of the MSPSS is 12–84, while each subscale ranges from 4 to 28. Higher scores indicate higher perceived levels of social support. Cronbach’s alpha coefficient for the Polish version of the MSPSS in this study was 0.91.

### Body esteem

Body esteem (BE) was evaluated using the validated Polish version of the Body‑Esteem Scale for Adolescents and Adults (BESAA) [26]. The Polish version of the BESAA includes 23 items comprising three subscales. The BE appearance subscale consists of 10 items referring to general feelings about one’s appearance (e.g., “I like how I look in photos”). The BE weight subscale consists of 8 items concerning satisfaction with one’s body weight (e.g., “I really like how much I weigh”). The five-item BE attribution subscale refers to beliefs about how one’s appearance is evaluated by others (e.g., “Other people think I look attractive”). Responses are provided on a five-point Likert scale: 1—“never”; 2—“seldom”; 3—“sometimes”; 4—“often”; and 5—“always”. For items 4, 7, 9, 11, 13, 17, 18, 19, and 21, reverse coding was applied as indicated by the authors [26]. The total score of the Polish version of the BESAA ranges from 23 to 155. BESAA subscale score ranges are as follows: BE appearance, 10–50; BE weight, 8–40; and BE attribution, 5–25. Higher scores indicate more positive attitudes and feelings toward one’s body and appearance. Cronbach’s alpha coefficient for the Polish version of the BESAA in this study was 0.94.

### Diet quality

Diet quality was assessed using the Dietary Habits and Nutrition Beliefs Questionnaire (KomPAN) [27]. The KomPAN questionnaire includes the major food groups in the Polish diet such as grain products (4 items); fruit, vegetables, legumes, and potatoes (4 items); dairy products (4 items); meat, fish, and eggs (5 items); fats (2 items); beverages (3 items); sweets; and other products (2 items). These 24 food items are divided into two groups: (1) food groups with a potentially beneficial effect on health; and (2) food groups with a potentially negative effect on health. Respondents reported habitual food consumption over the previous 12 months using a six-point scale: 1—“never”; 2—“1–3 times per month”; 3—“once per week”; 4—“a few times per week”; 5—“once per day”; and 6—“a few times per day”. These six responses were converted into numerical values expressed as frequency of consumption per day: 0.00; 2—0.06; 3—0.14; 4—0.50; 5—1.00; and 6—2.00, respectively [28].

Three dietary indices were calculated: 1—“Pro-Healthy Diet Index” (pHDI), covering 10 food groups with a potentially beneficial effect on health; 2—“Non-Healthy Diet Index” (nHDI), including 14 food groups with a potentially negative effect on health; and 3—“Diet Quality Index” (DQI), based on the consumption of all 24 food groups included in the pHDI and nHDI. To standardize the pHDI and nHDI ranges and facilitate interpretation, the sum of daily food consumption frequencies was expressed on a scale from 0 to 100 points. Scores of 0–33 indicate low intensity, 34–66 indicate medium intensity, and 67–100 indicate high intensity of beneficial (pHDI) or harmful (nHDI) dietary characteristics. The DQI was calculated as the sum of all positive components of the pHDI and all negative components of the nHDI. Weighted scores were applied, allowing the contribution of the 10 pHDI components to be equivalent to that of the 14 nHDI components. The DQI ranges from −100 to 100 points. Scores from −100 to −26 indicate a high intensity of nonhealthy dietary characteristics (predicting a harmful effect of diet on health); − 25 to 25 indicate low intensity of nonhealthy and pro-healthy dietary characteristics (predicting a neutral effect); and 26–100 indicate high intensity of pro-healthy dietary characteristics (predicting a beneficial effect) [28]. Cronbach’s alpha coefficient for the KomPAN in this study was 0.80.

### Sociodemographic characteristics

Sociodemographic characteristics were assessed using questions regarding: biological sex (“female”; “male”); age (in years); place of residence (“village”; “town below 20.000 inhabitants”; “town between 20.000 and 100.000 inhabitants”; “city over 100.000 inhabitants”); current stage of education (“high school”; “university”); education program (“class with a gastronomic education program”; “class with a comprehensive education program”; “nutrition-related major”; “non-nutrition-related major”); and high school grade or year of study.

### Anthropometric data

High school and university students self-reported their body weight and height. Body mass index (BMI) was calculated as body weight in kilograms divided by height in meters squared (kg/m^2^). According to the World Health Organization (WHO) criteria [29], BMI was used to classify students into four nutritional status categories: underweight, normal weight, overweight, and obese. For individuals aged ≥ 20 years were defined as underweight (BMI < 18.5 kg/m^2^), normal weight (BMI 18.5–24.9 kg/m^2^), overweight (BMI 25.0–29.9 kg/m^2^), and obesity (BMI ≥ 30.0 kg/m^2^) [29]. For participants aged 19 years and younger, BMI was assessed using age- and sex-specific WHO *z*-scores [30]. Underweight was defined as a BMI *z*-score below − 2 standard deviations (SD), normal weight as a *z*-score between − 2 SD and + 1 SD, overweight as a *z*-score above + 1 SD, and obesity as a *z*-score above + 2 SD [30].

### Statistical analysis

The normality of the distribution of continuous variables was assessed using the Shapiro–Wilk test, and homogeneity of variances was examined using Levene’s test. Categorical variables were presented as percentages (%) and numbers (*N*) of occurrences within the sample. As the distributions of all variables deviated from normality, nonparametric tests were performed. Accordingly, continuous variables were expressed as the median (Me) and interquartile range (IQR). Differences in continuous variables between two independent groups were analyzed using the Mann–Whitney U test, whereas differences among multiple independent groups were examined using the Kruskal–Wallis rank ANOVA followed by Dunn’s post hoc test. Differences between independent categorical variables were verified using the Pearson Chi-squared test.

Associations between ON risk and perceived social support, BE, and diet quality were analyzed using quantile regression analysis, including three quantiles: the first (Q_1_), second (Q_2_), and third (Q_3_). Q_1_ corresponds to the 25th percentile and represents the value below which 25% of the data fall. Q_2_ represents the 50th percentile (median), indicating that 50% of the data fall below this value, whereas Q_3_ corresponds to the 75th percentile, below which 75% of the data fall. ON risk, measured by the PL-DOS total score and treated as a continuous variable, was included in the models as the dependent variable. Independent variables included social support (continuous; scores), BE (continuous; scores), and the pHDI, nHDI, and DQI scores (continuous; scores). This method was selected because quantile regression does not require normality of the distribution of residuals, homoscedasticity, or linear relationships between the dependent and independent variables. In addition, quantile regression enables the estimation of associations at different points in the outcome distribution (Q1—25th percentile, Q2—median, Q3—75th percentile), allowing identification of factors associated with lower, median, and higher levels of ON risk. The parameter coefficient (*β*) with a 95% confidence interval (CI) and standard error (SE) was reported. The two models explained 15% of the variance in the dependent variable for models 1 and 2 (pseudo-*R*^2^ = 0.15 for both), whereas model 3 explained 19% of the variance (pseudo-*R*^2^ = 0.19). For all analyses, a *p* < 0.05 was considered statistically significant. Statistical analyses were performed using Statistica software, version 13.3 PL (StatSoft Inc., Tulsa, OK, USA; StatSoft, Kraków, Poland), and IBM SPSS Statistics for Windows, version 29.0 (IBM Corp., Armonk, NY, USA).

Effect sizes and post hoc power analyses were calculated using G*Power software version 3.1.9.7 (Heinrich-Heine-Universität Düsseldorf, Düsseldorf, Germany). As different statistical tests require different effect size measures, appropriate indices were applied: Glass’s rank bivariate correlation coefficient (rg) for the Mann–Whitney U test; epsilon squared (*ε*^2^) for the Kruskal–Wallis rank ANOVA; phi coefficient (*φ*) for 2 × 2 Pearson Chi-squared tests; and Cramer’s V coefficient (*V*) for more complex contingency tables. For Glass’s rank bivariate correlation coefficient, the phi coefficient, and Cramer’s V, values of 0.10 indicate a small effect, 0.30—a medium effect, and 0.50—a large effect. For epsilon squared, values of 0.01 indicate a small effect, 0.06—a medium effect, and 0.14—a large effect [31].

## Results

### Sample characteristics

The final sample comprised 261 high school and university students, including 64.8% females and 35.2% males. The median age of all students was 18.0 years (IQR = 1.0). High school students accounted for 54.0% of the participants, while 46.0% were university students. Among high school students, 31.4% were enrolled in classes with a gastronomic education program and 22.6% in a comprehensive education program. Nutrition-related majors were represented by 22.6% of participants, including fields such as Dietetics and Human Nutrition and Food Evaluation. The remaining 23.4% were students of non-nutrition-related majors, including Sociology. The majority of participants resided in a large city (37.5%). The median BMI was 21.5 kg/m^2^ (IQR = 4.5) (Table 1).

**Table 1:** Sociodemographic characteristics of the total study sample (*N* = 261)

Variables	*N*	%
Biological sex		
Female	169	64.8
Male	92	35.2
Age in years; Me (IQR)	18.0 (1.0)	
Place of residence *N* (%)		
Village	60	22.9
Town < 20.000 inhabitants	42	16.1
Town between 20.000 and 100.000 inhabitants	61	23.4
City > 100.000 inhabitants	98	37.5
Current stage of education *N* (%)		
High school	141	54.0
University	120	46.0
Education profile *N* (%)		
Classes with a gastronomic education program	82	31.4
Classes with a comprehensive education program	59	22.6
Nutrition-related majors	59	22.6
Non-nutrition-related majors	61	23.4
BMI in kg/m^2^; Me (IQR)	21.5 (4.5)	
BMI categories *N* (%)		
Underweight	10	3.8
Normal weight	207	79.3
Overweight	28	10.7
Obesity	16	6.1

*N* number of participants, *%* sample percentage, *Me* median, *IQR* interquartile range, *BMI* body mass index

### Biological sex differences

The risk of developing ON was observed in 18.0% of students, while 8.4% were characterized by the presence of ON. Females exhibited higher total PL-DOS scores (*p* = 0.004; rg = 0.41) and higher scores on its subscales, including negative feelings (*p* < 0.001; rg = 0.59) and social exclusion (*p* = 0.035; rg = 0.34), compared to males. A greater proportion of females than males demonstrated either a risk of developing ON or the presence of ON (*p* = 0.015; *V* = 0.18). Females also reported higher total perceived social support (*p* < 0.001; rg = 0.55), as well as higher social support from friends (*p* = 0.003; rg = 0.39) and significant others (*p* < 0.001; rg = 0.69), than males. Compared to males, females showed higher BE attribution scores (*p* = 0.030; rg = 0.27), whereas males exhibited higher BE weight scores than females (*p* = 0.003; rg = 0.39). Males demonstrated higher nHDI scores than females (*p* < 0.001; rg = 0.51), and a greater proportion of males than females were characterized by a medium level of nHDI (*p* = 0.002; *φ* = 0.19). Females exhibited higher DQI scores than males (*p* = 0.005; rg = 0.37). No significant differences between females and males were observed for the PL-DOS subscale importance of healthy nutrition, social support from family, BE total score, BE appearance, pHDI scores, or pHDI and DQI levels. None of the assessed students achieved high nHDI scores; therefore, this category was omitted from all subsequent tables (Table 2).

**Table 2:** Risk of orthorexia nervosa, perceived social support, body esteem, and diet quality across biological sex groups (*N* = 261)

Variables	Total sample	Female	Male	U/Chi^2^	Df	*V*/rg/*φ*	*p*-value
	*N* = 261	*N* = 169	*N* = 92				
Risk of orthorexia nervosa score; Me (IQR)							
PL-DOS total	21.0 (7.0)	22.0 (7.0)	20.0 (6.0)	2.81^●^	98	0.41^▲^	**0.004**
PL-DOS negative feelings	7.0 (3.0)	7.0 (4.0)	6.0 (2.0)	4.25^●^	98	0.59^▲^	** < 0.001**
PL-DOS social exclusion	4.0 (3.0)	5.0 (3.0)	4.0 (2.0)	2.11^●^	98	0.34^▲^	**0.035**
PL-DOS importance of health nutrition	10.0 (3.0)	10.0 (3.0)	10.0 (3.0)	0.42^●^	98	0.09^▲^	0.671
Risk of orthorexia nervosa groups *N* (%)							
Absence of orthorexia nervosa risk	192.0 (73.6)	115.0 (68.1)	77.0 (83.7)	8.42^●●^	2	0.18^▲▲^	**0.015**
Risk of developing orthorexia nervosa	47.0 (18.0)	35.0 (20.7)	12.0 (13.0)				
Presence of orthorexia nervosa	22.0 (8.4)	19.0 (11.2)	3.0 (3.3)				
Perceived social support; Me (IQR)							
Social support total	69.0 (18.0)	71.0 (14.0)	61.0 (21.5)	4.03^●^	98	0.55^▲^	** < 0.001**
Social support from family	22.0 (9.0)	22.0 (9.0)	20.0 (9.0)	1.83^●^	98	0.23^▲^	0.067
Social support from friends	24.0 (7.0)	24.0 (6.0)	22.0 (6.5)	3.02^●^	98	0.39^▲^	**0.003**
Social support from significant others	24.0 (8.0)	26.0 (6.0)	22.0 (8.5)	4.82^●^	98	0.69^▲^	** < 0.001**
Body esteem; Me (IQR)							
Body esteem total	66.0 (25.0)	65.0 (24.0)	67.5 (29.5)	−1.56^●^	98	0.21^▲^	0.118
Body esteem appearance	28.0 (14.0)	27.0 (13.0)	29.0 (14.0)	−1.87^●^	98	0.26^▲^	0.061
Body esteem weight	23.0 (13.0)	22.0 (12.0)	25.0 (9.0)	−2.99^●^	98	0.39^▲^	**0.003**
Body esteem attribution	15.0 (6.0)	15.0 (5.0)	14.0 (6.5)	2.17^●^	98	0.27^▲^	**0.030**
pHDI score; Me (IQR)	21.6 (15.0)	21.3 (16.1)	21.8 (14.3)	0.42^●^	98	0.00^▲^	0.672
Level pHDI *N* (%)							
Low	218 (83.5)	140.0 (82.8)	78.0 (84.8)	2.20^●●^	2	0.09^▲▲^	0.332*
Medium	42 (16.1)	29.0 (17.2)	13.0 (14.1)				
High	1 (0.4)	0.0 (0.0)	1.0 (1.1)				
nHDI score; Me (IQR)	15.6 (10.6)	14.6 (9.1)	17.9 (15.8)	−3.64^●^	98	0.51^▲^	** < 0.001**
Level of nHDI *N* (%)							
Low	240 (92.0)	162.0 (95.9)	78.0 (84.8)	9.88^●●^	1	0.19^▲▲▲^	0.002*
Medium	21 (8.0)	7.0 (4.1)	14.0 (15.2)				
DQI score; Me (IQR)	4.5 (17.6)	5.8 (16.3)	1.6 (16.7)	2.80^●^	98	0.37^▲^	**0.005**
Level of DQI *N* (%)							
High intensity of nHDI	2 (0.8)	2.0 (1.2)	0.0 (0.0)	4.11^●^	2	0.13^▲▲^	0.128*
Low intensity of nHDI and pHDI	241 (92.3)	152.0 (89.9)	89.0 (96.7)				
High intensity of pHDI	18 (6.9)	15.0 (8.9)	3.0 (3.3)				

*N* number of participants, *%* sample percentage, *Me* median, *IQR* interquartile range, *Df* degrees of freedom

^*^p-value corresponds to the overall Pearson’s Chi-square test for each variable; significance set at *p* < 0.05; bold values ​​indicate statistical significance

^●^U-Mann–Whitney test (U)

^●●^Pearson Chi-square test (Chi^2^)

^▲^Glass’s rank bivariate correlation coefficient (rg)

^▲▲^Cramer’s V coefficient (*V*)

^▲▲▲^Phi coefficient (*φ*)

### Education stage and program differences

University students from nutrition-related majors, compared with those from non-nutrition-related majors, demonstrated higher total PL-DOS scores (*p* = 0.047; *ε*^2^ = 0.19) and higher scores on the importance of healthy nutrition subscale (*p* = 0.004; *ε*^2^ = 0.22). University students from nutrition-related majors also reported higher total perceived social support (*p* = 0.016; *ε*^2^ = 0.18) and higher social support from family (*p* = 0.027; *ε*^2^ = 0.16) than high school students from classes with a gastronomic education program. University students from non-nutrition-related majors exhibited lower pHDI scores than university students from nutrition-related majors and high school students from gastronomic education programs (*p* = 0.004; *ε*^2^ = 0.20). High school students from comprehensive education programs, compared with university students from nutrition-related majors, demonstrated higher nHDI scores (*p* = 0.006; *ε*^2^ = 0.24). University students from nutrition-related majors exhibited higher DQI scores than those from non-nutrition-related majors and high school students from gastronomic education programs (*p* = 0.001; *ε*^2^ = 0.24). No significant differences were found across education stage and program for the PL-DOS subscales, negative feelings and social exclusion, ON risk groups, social support from friends and significant others, BE total score and its three subscales, or pHDI, nHDI, and DQI levels (Table 3).

**Table 3:** Risk of orthorexia nervosa, perceived social support, body esteem, and diet quality across current education stage and program (*N* = 261)

Variables	Classes with a gastronomic education program	Classes with a comprehensive education program	Nutrition-related majors	Non-nutrition-related majors	H/Chi^2^	*V*/*ε*^2^	Df	*p*-value
	*N* = 59	*N* = 82	*N* = 59	*N* = 61				
Risk of orthorexia nervosa; Me (IQR)								
PL-DOS total	20.0 (6.0)^a,b,c,d^	22.0 (6.0)^a,b,c,d^	22.0 (9.0)^a,b,c^	21.0 (7.0)^a,b,d^	7.95^●^	0.19^▲^	3	**0.047**
PL-DOS negative feelings	6.0 (3.0)	7.0 (4.0)	7.0 (3.0)	6.0 (3.0)	3.85^●^	0.12^▲^	3	0.278
PL-DOS social exclusion	4.0 (3.0)	5.0 (2.0)	5.0 (3.0)	4.0 (3.0)	6.46^●^	0.14^▲^	3	0.091
PL-DOS importance of health nutrition	10.0 (2.0)^a,b,c,d^	10.0 (4.0)^a,b,c,d^	11.0 (3.0)^a,b,c^	9.0 (3.0)^a,b,d^	13.18^●^	0.22^▲^	3	**0.004**
Risk of orthorexia nervosa groups *N* (%)								
Absence of orthorexia nervosa risk	46.0 (78.0)	58.0 (70.7)	39.0 (66.1)	49.0 (80.3)	9.58^●●^	0.14^▲▲^	6	0.143
Risk of developing orthorexia nervosa	12.0 (20.3)	15.0 (18.3)	11.0 (18.6)	9.0 (14.8)				
Presence of orthorexia nervosa	1.0 (1.7)	9.0 (11.0)	9.0 (15.3)	3.0 (4.9)				
Perceived social support; Me (IQR)								
Social support total	62.0 (20.0)^a,b,d^	70.0 (16.0)^a,b,c,d^	72.0 (14.0)^b,c,d^	66.0 (25.0)^a,b,c,d^	10.29^●^	0.18^▲^	3	**0.016**
Social support from family	20.0 (10.0)^a,b,d^	22.0 (9.0)^a,b,c,d^	24.0 (6.0)^b,c,d^	20.0 (9.0)^a,b,c,d^	9.17^●^	0.16^▲^	3	**0.027**
Social support from friends	23.0 (6.0)	24.0 (7.0)	24.0 (6.0)	23.0 (9.0)	5.83^●^	0.12^▲^	3	0.120
Social support from significant others	22.0 (8.0)	25.0 (6.0)	25.0 (6.0)	25.0 (10.0)	7.51^●^	0.16^▲^	3	0.057
Body esteem; Me (IQR)								
Body esteem total	66.0 (22.0)	65.0 (25.0)	68.0 (29.0)	64.0 (31.0)	0.36^●^	0.05^▲^	3	0.948
Body esteem appearance	28.0 (12.0)	29.0 (15.0)	29.0 (13.0)	26.0 (15.0)	0.27^●^	0.03^▲^	3	0.965
Body esteem weight	24.0 (13.0)	23.5 (12.0)	23.0 (13.0)	21.0 (15.0)	0.59^●^	0.04^▲^	3	0.898
Body esteem attribution	15.0 (5.0)	15.0 (7.0)	15.0 (5.0)	15.0 (6.0)	0.74^●^	0.06^▲^	3	0.863
pHDI score; Me (IQR)	19.9 (14.3)^a,b,c^	23.6 (14.4)^a,b,c,d^	24.1 (15.0)^a,b,c^	16.6 (13.6)^b,d^	13.16^●^	0.20^▲^	3	**0.004**
Level pHDI *N* (%)								
Low	49.0 (83.1)	64.0 (78.1)	49.0 (83.1)	56.0 (91.8)	8.27^●●^	0.13^▲▲^	6	0.218*
Medium	9.0 (15.2)	18.0 (21.9)	10.0 (16.9)	5.0 (8.2)				
High	1.0 (1.7)	0.0 (0.0)	0.0 (0.0)	0.0 (0.0)				
nHDI score; Me (IQR)	15.6 (11.6)^a,b,c,d^	17.9 (13.0)^a,b,d^	13.1 (6.4)^a,c,d^	17.0 (9.1)^a,b,c,d^	12.54^●^	0.24^▲^	3	**0.006**
Level of nHDI *N* (%)								
Low	53.0 (89.8)	73.0 (89.0)	58.0 (98.3)	56.0 (91.8)	5.83^●●^	0.13^▲▲^	3	0.209*
Medium	6.0 (10.2)	9.0 (11.0)	1.0 (1.7)	5.0 (8.2)				
DQI score; Me (IQR)	3.1 (14.4)^a,b,d^	6.0 (17.6)^a,b,c,d^	11.5 (17.9)^b,c^	−0.4 (18.1)^a,b,d^	16.13^●^	0.24^▲^	3	**0.001**
Level of DQI *N* (%)								
High intensity of nHDI	0.0 (0.0)	1.0 (1.2)	0.0 (0.0)	1.0 (1.6)	3.77^●●^	0.08^▲▲^	6	0.707*
Low intensity of nHDI and pHDI	55.0 (93.2)	76.0 (92.7)	53.0 (89.8)	57.0 (93.4)				
High intensity of pHDI	4 (6.8)	5 (6.1)	6 (10.2)	3 (5.0)				

*N* number of participants, *%* sample percentage, *Me* median, *IQR* interquartile range, *Df* degrees of freedom, *pHDI* Pro-Healthy Diet Index, *nHDI* Non-Healthy Diet Index, *DQI* Diet-Quality Index

^a,b,c,d^Means differ statistically significantly at *p* < 0.05; bold values ​​indicate statistical significance

^*^p-value corresponds to the overall Pearson’s Chi-square test for each variable

^●^Kruskal–Wallis rank ANOVA test (H), and the Dunn post hoc test

^●●^Pearson Chi-square test (Chi^2^)

^▲^epsilon squared (*ε*^2^)

^▲▲^Cramer’s V coefficient (*V*)

### BMI group differences

Students with normal weight compared with those with obesity exhibited higher BE total scores (*p* < 0.001; *ε*^2^ = 0.33), BE appearance scores (*p* = 0.014; *ε*^2^ = 0.22), and BE weight scores (*p* < 0.001; *ε*^2^ = 0.32). Students with obesity demonstrated lower BE attribution than those with underweight and normal weight (*p* < 0.001; *ε*^2^ = 0.38). Most students with normal weight were characterized by a medium level of pHDI (*p* = 0.012; *V* = 0.18). No significant differences were observed across BMI groups for PL-DOS total scores and subscales, ON risk groups, total perceived social support and its subscales, or pHDI scores, or nHDI and DQI scores and levels (Table 4).

**Table 4:** Risk of orthorexia nervosa, perceived social support, body esteem, and diet quality across the BMI groups (*N* = 261)

Variables	Underweight	Normal weight	Overweight	Obesity	H/Chi^2^	*V*/*ε*^2^	Df	*p*-value
	*N* = 10	*N* = 207	*N* = 28	*N* = 16				
Risk of orthorexia nervosa; Me (IQR)								
PL-DOS total	21.0 (7.0)	21.0 (8.0)	21.0 (6.0)	20.5 (5.5)	0.55^●^	0.05^▲^	3	0.907
PL-DOS negative feelings	7.0 (4.0)	7.0 (4.0)	6.5 (4.0)	7.0 (3.0)	0.58^●^	0.05^▲^	3	0.902
PL-DOS social exclusion	4.0 (3.0)	4.0 (3.0)	4.5 (2.5)	4.5 (3.0)	0.15^●^	0.01^▲^	3	0.985
PL-DOS importance of health nutrition	11.0 (4.0)	10.0 (3.0)	10.5 (4.0)	9.0 (2.0)	4.72^●^	0.13^▲^	3	0.193
Risk of orthorexia nervosa groups *N* (%)								
Absence of orthorexia nervosa risk	6.0 (60.0)	152.0 (73.4)	21.0 (75.0)	13.0 (81.3)	3.43	0.08	6	0.754
Risk of developing orthorexia nervosa	2.0 (20.0)	37.0 (17.9)	5.0 (17.9)	3.0 (18.8)				
Presence of orthorexia nervosa	2.0 (20.0)	18.0 (8.7)	2.0 (7.1)	0.0 (0.0)				
Perceived social support; Me (IQR)								
Social support total	68.0 (32.0)	70.0 (17.0)	67.5 (27.0)	60.5 (18.5)	3.49^●^	0.08^▲^	3	0.322
Social support from family	23.5 (10.0)	22.0 (8.0)	19.0 (11.0)	17.0 (10.5)	6.97^●^	0.17^▲^	3	0.073
Social support from friends	21.5 (10.0)	24.0 (7.0)	23.0 (7.5)	22.5 (6.0)	0.96^●^	0.05^▲^	3	0.811
Social support from significant others	25.5 (12.0)	24.0 (7.0)	24.0 (11.0)	25.5 (7.5)	0.96^●^	0.09^▲^	3	0.810
Body esteem; Me (IQR)								
Body esteem total	58.5 (29.0)^a,b,c,d^	67.0 (28.0)^a,b,c^	59.5 (26.0)^a,b,c,d^	49.0 (20.5)^a,c,d^	20.78^●^	0.33^▲^	3	** < 0.001**
Body esteem appearance	25.5 (17.0)^a,b,c,d^	29.0 (14.0)^a,b,c^	25.0 (15.5)^a,b,c,d^	22.0 (11.0)^a,c,d^	10.54^●^	0.22^▲^	3	**0.014**
Body esteem weight	21.5 (10.0)^a,b,c,d^	24.0 (11.0)^a,b,c^	21.0 (11.5)^a,b,c,d^	16.0 (10.5)^a,c,d^	20.51^●^	0.32^▲^	3	** < 0.001**
Body esteem attribution	17.0 (6.0)^a,b,c^	15.0 (6.0)^a,b,c^	14.0 (4.5)^a,b,c,d^	9.0 (7.0)^c,d^	25.19^●^	0.38^▲^	3	** < 0.001**
pHDI score; Me (IQR)	21.7 (16.8)	20.5 (15.2)	23.7 (8.7)	20.7 (15.8)	1.75^●^	0.06^▲^	3	0.626
Level pHDI *N* (%)								
Low	8.0 (80.0)	172.0 (83.1)	25.0 (89.3)	13.0 (81.3)	16.27^●●^	0.18^▲▲^	6	0.012*
Medium	2.0 (20.0)	35.0 (16.9)	3.0 (10.7)	2.0 (12.5)				
High	0.0 (0.0)	0.0 (0.0)	0.0 (0.0)	0.0 (0.0)				
nHDI score; Me (IQR)	12.2 (11.2)	15.8 (9.2)	15.8 (12.5)	14.0 (19.5)	0.91^●^	0.05^▲^	3	0.824
Level of nHDI *N* (%)								
Low	9.0 (90.0)	190.0 (91.8)	26.0 (92.9)	15.0 (93.8)	0.16^●●^	0.02^▲▲^	3	0.984*
Medium	1.0 (10.0)	17.0 (8.2)	2.0 (7.1)	1.0 (6.3)				
DQI score; Me (IQR)	5.8 (20.7)	4.3 (17.5)	5.9 (18.3)	0.7 (23.0)	0.50^●^	0.04^▲^	3	0.918
Level of DQI *N* (%)								
High intensity of nHDI	0.0 (0.0)	2.0 (0.9)	0.0 (0.0)	0.0 (0.0)	6.06^●●^	0.11^▲▲^	6	0.417*
Low intensity of nHDI and pHDI	8.0 (80.0)	191.0 (92.3)	28.0 (100.0)	14.0 (87.5)				
High intensity of pHDI	2.0 (20.0)	14.0 (6.8)	0.0 (0.0)	2.0 (12.5)				

*N* number of participants, *%* sample percentage, *Me* median, *IQR* interquartile range, *Df* degrees of freedom, *BMI* body mass index, *pHDI* Pro-Healthy Diet Index, *nHDI* Non-Healthy Diet Index, *DQI* Diet-Quality Index

^*^p-value corresponds to the overall Pearson’s Chi-square test for each variable

^a,b,c,d^Means differ statistically significantly at *p* < 0.05; bold values ​​indicate statistical significance

^●^Kruskal–Wallis Rank ANOVA test (H), and the Dunn post hoc test

^●●^Pearson Chi-square test (Chi^2^)

^▲^epsilon squared (*ε*^2^)

^▲▲^Cramer’s V coefficient (*V*)

### Quantile regression analysis of factors associated with ON risk

An increased risk of ON was associated with lower social support from friends, lower BE weight scores, and lower nHDI scores across all three models. In addition, higher pHDI scores were associated with an increased risk of ON across all three models. A higher BE attribution score was associated with an increased risk of ON only in model 3 (*β* = 0.33; *p* = 0.002) (Table 5).

**Table 5:** Quantile regression analysis of orthorexia nervosa risk in the total sample (*N* = 261)

Parameter	Model 1 (Q_1_ = 0.25)					Model 2 (Q_2_ = 0.50)					Model 3 (Q_3_ = 0.75)				
	*β*	SE	95% CI	Df	*p*-value	*β*	SE	95% CI	Df	*p*-value	*β*	SE	95% CI	Df	*p*-value
Social support from friends	−0.24	0.11	−0.45; −0.03	252	**0.023**	−0.18	0.09	−0.35; −0.01	252	**0.035**	−0.15	0.09	−0.33; 0.03	252	**0.104**
Body esteem weight	−0.24	0.09	−0.43; −0.06	252	**0.011**	−0.25	0.08	−0.40; −0.11	252	** < 0.001**	−0.28	0.08	−0.44; −0.11	252	** < 0.001**
Body esteem attribution	0.09	0.12	−0.14; 0.34	252	0.417	0.14	0.09	−0.05; 0.33	252	0.140	0.33	0.10	0.13; 0.54	252	**0.002**
pHDI score	0.09	0.05	0.01; 0.17	252	**0.028**	0.13	0.03	0.07; 0.19	252	** < 0.001**	0.12	0.04	0.05; 0.19	252	** < 0.001**
nHDI score	−0.19	0.04	−0.28; −0.09	252	** < 0.001**	−0.22	0.04	−0.29; 0.14	252	** < 0.001**	−0.11	0.05	−0.21; −0.02	252	**0.023**

*β* coefficient of parameter, *SE* standard error, *95% CI* 95% confidence interval, *Df* degrees of freedom, *Q* quantile, *pHDI* Pro-Healthy Diet Index, *nHDI* Non-Healthy Diet Index

Significance set at *p* < 0.05; bold values ​​indicate statistical significance

## Discussion

The current study evaluated the associations between the level of orthorexia nervosa risk, perceived social support, body esteem, and diet quality according to biological sex, current education stage and program, and BMI groups.

The presence of ON among the studied high school and university students was 8.4%, while the risk of developing ON was more than twice as high (18.0%). In previous studies using the DOS, the presence of ON ranged from 1.5 to 10.5% [32, 33], whereas the risk of developing ON ranged from 4.9 to 12.4% [24, 34]. Thus, the findings of the current study are consistent with earlier reports regarding the presence of ON, although the observed risk of developing ON was slightly higher. To date, the DOS has been used in various language versions, with different cut-off specifications, which, together with cultural factors, may partly explain variations in prevalence estimates [24, 32–34].

Interestingly, on the social exclusion subscale of the PL-DOS, high school and university students scored very low (score of 4, with a possible range of 3–12) compared with the other subscales. EDs, including AN, bulimia nervosa (BN), and binge eating disorder (BED), have a profound impact on social relationships [35]. Individuals affected by these disorders often avoid situations in which food or eating plays an important role, such as social gatherings, group meals, or family celebrations [36]. This avoidance may lead to social withdrawal, conflicts with relatives, and difficulties in establishing and maintaining relationships [37]. Therefore, it may be assumed that the studied high school and university students did not experience food- or eating-related problems severe enough to result in a high level of social exclusion. Nevertheless, an increase in ON risk was associated with lower social support from friends across all three models (Q1–Q3), whereas support from family and significant others was not a significant predictor. This finding is consistent with the literature emphasizing the role of peer support during adolescence and early adulthood [38]. Research indicates that during this developmental period, relationships with friends become increasingly important and may exert a stronger influence on health behaviors than support from family or significant others (e.g., teachers, mentors, or distant acquaintances) [39]. A lack of social support from friends may be associated with greater susceptibility to compensatory behaviors, including restrictive eating behaviors [36].

Importantly, the total PL-DOS score was positively associated with pHDI and negatively associated with nHDI in all three models (Q1–Q3), highlighting the complex nature of ON [17]. Individuals with ON tend to select foods they perceive as healthy [1] and avoid fatty or sweetened foods, nonorganic products, and foods containing pesticides, herbicides, genetically modified ingredients, artificial additives, or other undesired components [40]. Consequently, they often eliminate certain foods or entire food groups [1]. Paradoxically, although individuals with ON report striving for healthier eating, such dietary patterns may result in micro- and macronutrient deficiencies [14]. This indicates that higher diet quality does not necessarily translate into optimal health [1]. Some researchers suggest that excessive adherence to dietary rules and reduced flexibility in food choices may, over time, decrease overall diet quality [41]. However, given the cross-sectional design of the present study, it cannot be determined whether the diets of high school and university students with ON would deteriorate over time.

Educational programs focused on health, nutrition, and food have also been associated with a higher ON risk [21, 22]. The present findings support this association, as students enrolled in nutrition-related majors exhibited a higher ON risk than students in other fields. Moreover, students in nutrition-related majors and high school students attending classes with a gastronomic education program demonstrated higher diet quality compared with their peers outside these programs. The higher diet quality observed among students engaged in health-, nutrition-, and food-related education may be explained by greater nutritional knowledge, awareness, and motivation to adopt healthy eating behaviors [16]. However, a potential paradox arises in that an intense interest in healthy eating combined with high nutritional knowledge may foster excessive control and dietary rigidity, ultimately leading to difficulties in maintaining flexible and balanced eating behaviors [42].

Biological sex also differentiated the ON risk in the current study, with females exhibiting a higher risk than males. Some previous studies have reported similar findings [43, 44], whereas others found no association between biological sex and ON risk [42]. Notably, females in the present study not only scored higher on the PL-DOS total score and the negative feelings subscale, but also on the social exclusion subscale. This finding is particularly interesting given that females reported higher total perceived social support, as well as higher support from friends and significant others, compared with males. Research suggests that although females are more likely than males to report having social support, they do not always perceive this support as adequate or effective [45]. EDs are often accompanied by feelings of being misunderstood by others [46], and despite the formal availability of social support, individuals with EDs may experience a sense of isolation [47]. Some researchers propose that a sense of social safety, rather than the mere presence of perceived or received support, may be more closely associated with lower ED symptom severity [48]. In addition, females are exposed to greater sociocultural pressure related to appearance and body weight control than males, which may heighten sensitivity to social evaluation and intensify feelings of otherness [49]. In the present study, this pattern was also evident, as females, despite reporting firmer beliefs regarding positive evaluation of their appearance by others, experienced lower satisfaction with their body weight than males.

Finally, self-esteem related to body weight and attribution was associated with ON risk in the present study. An increased ON risk was associated with lower body weight-related self-esteem across all models (Q1–Q3) and with higher attribution-related self-esteem in the highest quantile (Q3). Early conceptualizations of ON suggested an absence of weight loss focus or negative body image, in contrast to AN and BN [1]. Although ON is defined primarily by concern for health and well-being rather than appearance or body weight [1], recent research has demonstrated associations between ON and drive for thinness, low self-esteem, poor body image, and social physique anxiety related to body dissatisfaction [8, 50]. These findings may suggest that some psychosocial aspects observed in individuals at ON risk resemble patterns previously reported in the AN literature, particularly those related to body image. However, as the present study did not directly assess symptoms of AN, no conclusions can be drawn about the broader overlap between these conditions. Nevertheless, existing evidence regarding the association between BMI and ON risk remains inconclusive, highlighting the need for further studies aimed at better distinguishing ON from AN [51], particularly given that BMI is a key criterion for determining AN severity [4, 5]. In the present study, no association was observed between BMI and ON risk; however, this finding should be interpreted with caution, given the self-reported nature of BMI and the limited sample sizes within some BMI categories. Furthermore, research suggests that a slim body is often socially associated with attributes such as strong willpower, self-control, attractiveness, intelligence, and success [52]. In the context of EDs, this may lead to a paradox in which individuals are socially rewarded for appearances that result from pathological behaviors [53].

### Strengths and limits

The strength of this study lies in the diversity of the sample with regard to the current education stage and program. The study included both high school and university students enrolled in programs related to human nutrition, food, and gastronomy, as well as programs unrelated to these fields. This design enabled a comparison between a group at high risk of ON [21, 22] and a group with a potentially lower risk. Moreover, to the best of our knowledge, this is the first study to simultaneously evaluate the associations between ON risk, perceived social support, BE, and diet quality using a reliable instrument such as the PL-DOS. An additional strength is the use of validated Polish versions of standardized questionnaires, including the PL-DOS, BESAA, and MSPSS, thereby enhancing the reliability and comparability of the findings. Nevertheless, the study has several limitations.

Data were collected exclusively from high school and university students; therefore, the findings cannot be generalized to the general population. The nonrandom selection of high schools and the university was based on accessibility, which may have introduced selection bias. Although identical procedures were applied across all recruitment points, the extended data-collection period may have introduced contextual differences in the timing of data collection that were not formally examined. In addition, there was a noticeable biological sex imbalance in the sample, with males representing 35.2% of participants, which limits the conclusions that can be drawn from comparisons between females and males. BMI was calculated using self-reported body weight and height, which may have resulted in inaccuracies due to reporting bias. Self-reported data can be influenced by sociodemographic factors, such as biological sex [54]. Furthermore, some subgroup analyses were based on relatively small sample sizes, which may have limited the precision of certain estimates. In addition, multiple subgroup comparisons and analyses involving several scales, subscales, and dietary indicators were conducted; therefore, the possibility of type I error cannot be excluded, and findings, particularly those with borderline statistical significance, should be interpreted with caution. It should also be noted that some of the applied instruments, particularly the PL-DOS and MSPSS, have been extensively validated in adult and young adult populations, aligning well with the university students included in this study. However, given the more limited evidence for their use in strictly adolescent samples, caution is warranted when interpreting findings for the youngest participants. In addition, the KomPAN questionnaire assesses food intake over the previous 12 months, which may not fully reflect recent eating changes typical for student populations. Moreover, the dietary indices used in this study (pHDI, nHDI, and DQI), although useful for assessing general diet quality, do not directly capture cognitive or behavioral aspects such as eating rigidity or rule-based eating, which are central features of ON. Additionally, the quantile regression models explained only 15.0% or 19.0% of the variance in the dependent variable. Finally, the cross-sectional design, with data collected at a single time point, does not allow for causal inferences regarding the observed associations.

## What is already known on this subject?

ON is characterized by an excessive preoccupation with healthy eating and rigid dietary rules and remains a controversial construct due to its overlap with established EDs, particularly AN. Although initially considered unrelated to weight and shape concerns, recent evidence indicates associations between ON risk, body dissatisfaction, lower self-esteem, and drive for thinness. Importantly, ON, similarly to other EDs, has been related to lower perceived social support, which may exacerbate maladaptive eating behaviors. Individuals at risk of ON tend to engage in progressive dietary restriction; however, findings regarding the relationship between orthorexia nervosa risk and diet quality remain inconsistent.

## What this study adds?

The findings of the present study indicate that ON risk is associated with body weight-related self-esteem, perceived social support from friends, and diet quality among adolescents and young adults. The absence of an association between ON risk and BMI indicates that future research should focus on identifying alternative indicators that differentiate ON from other EDs. Furthermore, education programs in health, nutrition, and food should incorporate more comprehensive content addressing the psychological aspects of eating and body image in order to reduce the risk of both ON and EDs. Social support from friends emerged as a crucial factor; therefore, prevention and intervention efforts should emphasize the development of high-quality peer relationships and the strengthening of social skills that promote healthy behaviors within friendship groups. Additionally, incorporating moderating variables such as the quality of social support and social safety into predictive models may provide a more nuanced understanding of ON risk. Longitudinal studies are also warranted to determine whether the initially high diet quality observed among individuals with ON deteriorates over time as a result of excessive restrictiveness and reduced dietary flexibility.

## Data Availability

All the data and syntaxes used for the analyses presented within the paper are stored at the open science repository, which is available at the link: https://doi.org/10.18150/L7PTIL.
